# The Medical News Visiting List for 1892

**Published:** 1892-01

**Authors:** 


THE MEDICAL NEWS VISITING LIST FOR 1892.—Published in four styles, by Lea Brothers & Company, Philadelphia Pa.

Weekly, dated for 30 patients, monthly, undated, for 120 patients per month; Perpetual undated, for 30 patients per week, per year, and perpetual undated, for 60 patients per week, per year (without text.) The first three styles contain, 2 pages of text and 176 pages of blanks. The 60 patients style consists of 256 pages of blanks. Wallet size, flexible leather cover, pocket, pencil and catheter scale. Price, any style, $1.25. Thumb lettered index, 25 cents extra.

This is a very neat, compact, useful visiting list, containing in text: Signs of Dentition, To find Day of Confinement, Thermo-metric Scales, Weights and Measures (ordinary and metric), Comparative Scales, Examination of Urine, Incompatibles, Artificial Respiration, Table of Eruptive Fevers, Poisons and Antidotes, Table of Doses, Therapeutic Remedies, Ligations of Arteries, with the blanks necessary for the use of a busy practitioner. K.

